# Bleeding of the Superior Vena Cava Due to an Iatrogenic Injury to It during the Ultrasound-Guided Central Venous Cannulation

**DOI:** 10.3390/medicina58020266

**Published:** 2022-02-10

**Authors:** Seong-Hoon Jung, Dae-Hwan Kim, Jeong-Eun Sohn

**Affiliations:** Department of Anesthesiology and Pain Medicine, Cheju Halla General Hospital, Jeju 63127, Korea; jujdmaster@naver.com (S.-H.J.); mystletain@naver.com (D.-H.K.)

**Keywords:** esophageal perforation, catheterization, central venous, ultrasonography, vena cava, superior, medical errors

## Abstract

Central venous cannulation (CVC) is a procedure that is frequently performed to facilitate resuscitation, nutritional support and long-term vascular access. It may often cause mechanical complications during placement of a cannula in association with the anatomical relationship with central veins. A 68-year-old man visited our medical institution with a chief complaint of foreign-body-induced esophageal perforation. This patient presented with bleeding of the superior vena cava due to an iatrogenic injury to it during the CVC in the right internal jugular vein. Our case indicates that it would be mandatory to insert a cannula at an optimal depth considering the anatomical relationship between the central veins during the CVC.

## 1. Introduction

The superior vena cava (SVC) is vulnerable to diverse types of damages. Most cases of SVC injuries are iatrogenic, which result from the placement of central venous catheters, insertion of pacemakers, stenting of SVC in SVC obstruction syndrome or placement of a filter in the SVC to prevent showering of emboli [1]. Several cases of erosion or rupture of SVC have been described in the literature, which lead to hemodynamic instability, hemothorax or pericardial tamponade, thus requiring prompt action to avoid cardiovascular collapse and death [2,3,4,5,6,7]. This is a potentially fatal complication causing sudden hemodynamic compromise associated with high mortality, for which an immediate surgical intervention is mandatory, unless recognized and treated promptly [8].

Central venous cannulation (CVC) is a procedure that is frequently performed to facilitate resuscitation, nutritional support and long-term vascular access. It may often cause mechanical complications during the placement of a cannula in association with the anatomical relationship with central veins [9].

We experienced a case of a 68-year-old man presenting with bleeding of the SVC due to an iatrogenic injury to it during the CVC in the right internal jugular vein (RIJV). Here, we report our case with a review of literature.

## 2. Case Presentations

A 68-year-old man visited our medical institution. On chest computed tomography (CT) scans, there were two radiopaque foreign bodies at the height of the carina in the middle esophagus. A chest CT also showed mediastinitis.This was accompanied by the presence of a small air shadow that is suggestive of perforation outside the esophagus.The patient was diagnosed with foreign body-induced esophageal perforation (EP).

At an operating room, general anesthesia was induced using remifentanil 0.05 μg/kg/min, propofol 80 mg and rocuronium 50 mg after preoxygenation with 100% oxygen at a rate of 5 L/min. Then, anesthesia was maintained using oxygen 1.5 L/min, air 1.5 L/min, sevoflurane 2 vol% and remifentanil 0.05 μg/kg/min. The CVC was planned for the RIJV using triple-lumen central venous catheterization set with blue flex tip (Blue FlexTip^®^ARROWg+ard Blue^®^ Catheter; Arrow International, Inc., Reading, PA, USA) when the patient intraoperatively had a bleeding. 

At an operating room, a resident at department of anesthesiology and pain medicineperformed ultrasound (US)-guided puncture of the RIJV. Then, we inserted an 18 g puncture needle-attached syringe in the RIJV at an angle of 30°. Following this, we aspirated dark non-pulsatile venous blood from the RIJV while advancing the GW in the RIJV at a depth of approximately 3 cm. On noticing that the depth of insertion reached approximately 30 cm, we planned to place a dilator in the RIJV and to replace the dilator with a catheter. Considering that the catheter and the GW were not well placed, however, we removed both. Then, we removed a puncture needle and then compressed the site of puncture using a gauze pad for 5 min again for hemostasis. The CVC was performed twice, but in vain.

The senior author performed the third session of the CVC for the right subclavian vein (RSV). We attempted to perform a puncture again using an 18 g puncture needle-attached syringe in the area 1 cm remote from the inferior lateral side of the junction between the medial and middle 1/3 of the clavicle. Then, we gradually advanced the needle below the clavicle towards the sternal notch. Nevertheless, we failed in performing a puncture for the RSV despite a sufficient depth of puncture. Then, we took appropriate measures against bleeding by placing two peripheral lines in both arms. 

**Figure 1:**
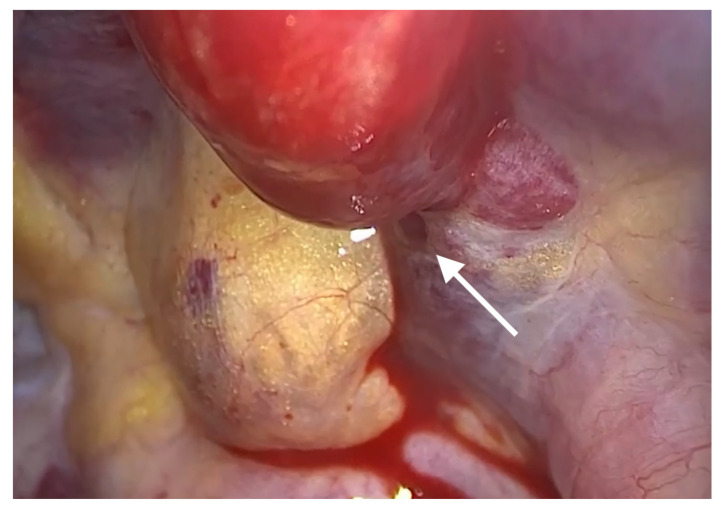
Bleeding of the superior vena cava (arrow) on video-assisted thoracoscopic surgery. Hematoma of the brachiocephalic vein is also seen.

**Figure 2:**
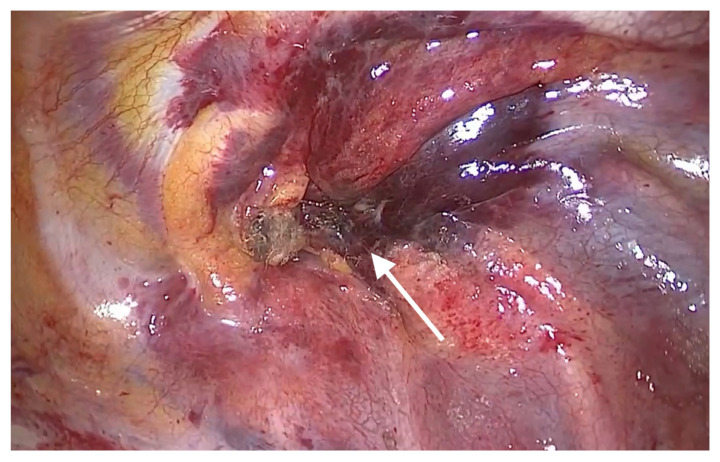
Intraoperative hemostasis at the site of perforation (arrow) of the superior vena cava.

After the insertion of a trocar in the fifth intercostal space on the midaxillary line and that of a thoracoscope, we noticed that bleeding and hematoma occurred in the SVC. The site of perforation was located in the extra-pericardial portion of the SVC.Even after the aspiration of blood at a volume of approximately 700 mL, bleeding was persistently present at the site of perforation in the SVC. This was accompanied by the presence of hematoma in the right brachiocephalic vein (Figure 1). The patient had a gradual decrease in blood pressure. Therefore, the patient received a continuous intravenous infusion of norepinephrine 0.05 μg/kg/min and the crystalloid solution at a rate of 600 mL/hr. For hemostasis, the site of bleeding in the SVC was compressed using a gauze pad for 20 min, followed by the use of an active absorbable collagen hemostat. On noticing that bleeding was discontinued, we performed surgery to remove foreign bodies from the esophagus (Figure 2).

The foreign body was found to be thethorn of a fish (Horsehead tilefish). Postoperatively, hemostasis and extubation were performed. Then, the patient was transferred to an intensive care unit (ICU). After a 7-day ICU hospitalization, the patient was transferred to a general ward and then discharged on postoperative day 24. The postoperative course was uneventful.

## 3. Discussion and Conclusions

The CVC can be performed via diverse veins according to a clinician’s preference, indications for it and a patient’s medical conditions [10]. The RIJV has been the preferred site for the CVC because it is easy to place a cannula in the RIJV by using the anatomical landmarks of the internal carotid artery (ICA) and the sternocleidomastoid muscle (SCM) [10]. Moreover, the RIJV follows a short and straight path to the SVC, which makes it easier to perform the CVC for the SVC. Therefore, the possibility of misplacement of a cannula in wrong vessels can be minimized [10,11]. This explains why we performed the first and second session of the CVC for the RIJV in our case.

It has been suggested that the CVC in the IJV might fail or cause complications in association with its anatomical variations [12]. There is a great variability in the frequency of anatomical variations of the IJV depending on a patient between the authors [12,13,14,15,16,17,18,19]. In our case, however, the patient had no anatomical variations of the RIJV.

However, little is known about the exact pathophysiologic mechanisms underlying the occurrence of an iatrogenic injury to the SVC during the CVC. Abdelke et al. reported that the tip of a cannula is often positioned in direct contact with the lateral wall of the SVC where the innominate vein forms a right angle to the SVC [20]. According to Robinson et al. and Oropello et al., a dilator can cause a direct injury to the wall of great vessels during CVC when it is inserted through the Seldinger technique [21,22]. In more detail, Oropello et al. noted that a dilator would cause an injury to the wall of great vessels if its length is greater than 8 cm. According to these authors, a relatively flexible J-tip GW would not be appropriate for inducing a route of insertion of a dilator that is inserted with a strong force at a relatively greater depth. It can therefore be inferred that bending of the J-tip GW might cause an injury to the wall of great vessels. Thus, they emphasized that the dilator should be solely used to dilate the skin, subcutaneous tissue and the wall of the target vein at an optimal depth of <8 cm [22].

**Figure 3:**
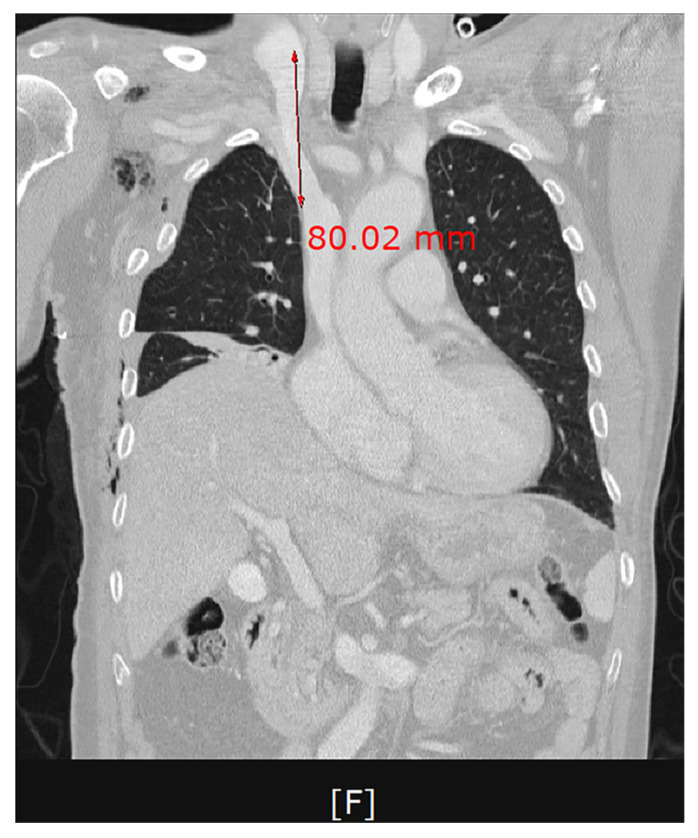
A frontal computed tomography scan of the chest. Approximately 80% of the length of a dilator corresponding to the distance between the site of puncture on the internal jugular vein and that on the superior vena cava.

We assumed that a dilator caused an injury to the wall of the SVC while being pushed deeply at the point of a crooked part in the first session of the CVC. We used a dilator with a length of 10.2 cm. Approximately 80% of the length of a dilator corresponded to the distance between the site of puncture on the internal jugular vein and that on the SVC (Figure 3). This is in agreement with a previous report that it would not be necessary to insert a dilator of ≥8 cm in length [22].

There are two traditional techniques for the CVC; these include the anatomical landmark (AL) technique and a US-guided one. To date, studies have been conducted in anesthetic, cardiovascular and ICU settings and then shown that US-guided CVC, via the IJV in particular, might be more beneficial for lowing the rates of complications and shortening the procedural time as compared with the AL technique [9]. In the current case, we performed the US-guided CVC. 

As shown in the current case, the patient sustained an iatrogenic injury to the SVC during the US-guided CVC. Another similar case has been described in the literature: the SVC perforation occurred during the CVC in a 17-year-old man. In this patient, Çelik B et al. performed a video-assisted thoracoscopic surgery with hemostasis. Thus, these authors emphasized the importance of postoperative management of an iatrogenic injury to the SVC due to the CVC [23].

In conclusion, our case indicates that it would be mandatory to insert a cannula at an optimal depth considering the anatomical relationship between the central veins during the CVC.

## Data Availability

The datasets of the current study are available from the corresponding author upon reasonable request.
